# In-hospital COVID-19 infection echocardiographic analysis: a Brazilian, tertiary single-centre experience

**DOI:** 10.1186/s12947-021-00265-y

**Published:** 2021-10-23

**Authors:** Marcelo Luiz Campos Vieira, Tania Regina Afonso, Alessandra Joslin Oliveira, Carolina Stangenhaus, Juliana Cardoso Dória Dantas, Lucas Arraes de França, Edgar Daminelo, Adriana Cordovil, Lara A. S. Martins, Rodrigo A. C. Meirelles, Rafael B. Piveta, Sérgio Barros-Gomes, Miguel O. D. Aguiar, Patrícia O. Roveri, Wércules A. Oliveira, Alessandro C. Lianza, Andrea P. L. Ponchirolli, Líria M. L. Silva, Rodrigo C. P. L. Costa, Cláudio H. Fischer, Samira Saady Morhy

**Affiliations:** Laboratory of Echocardiography, Hospital Israelita Albert Einstein, Av. Albert Einstein, 627/701, São Paulo, ZIP code: 05651-901 Brazil

**Keywords:** COVID-19, Echocardiography, Mortality, pulmonary thromboembolism, Renal failure

## Abstract

**Background:**

Information is lacking concerning in-hospital echocardiography analysis of COVID-19 infection in Brazil. We evaluated echocardiographic parameters to predict a composite endpoint of mortality, pulmonary thromboembolism or acute renal failure.

**Methods:**

A prospective full echocardiographic study of consecutive patients hospitalized with COVID-19, single tertiary centre in Brazil. We correlated echocardiographic findings to biomarkers, clinical information, thoracic tomography, and in-hospital composite endpoint of mortality, pulmonary thromboembolism or renal failure.

**Results:**

One hundred eleven patients from March to October 2020, 67 ± 17 years, 65 (58.5%) men, death was observed in 21/111 (18.9%) patients, 48 (43%) required mechanical ventilation, myocardial infarction occurred in 10 (9%), pulmonary thromboembolism in 7 (6.3%) patients, haemodialysis was required for 9 (9.8%). Echocardiography was normal in 51 (46%) patients, 20 (18%) presented with decreased left ventricle ejection, 18 (16.2%) had abnormal left ventricle global longitudinal strain, 35 (31%) had diastolic dysfunction, 6 (5.4%) had an E/e’ratio > 14, 19 (17.1%) presented with right ventricle dilated/dysfunction, 31 (28%) had pericardial effusion. The echocardiographic parameters did not correlate with mortality, biomarkers, clinical events. Tricuspid velocity was related to the composite endpoint of mortality, pulmonary thromboembolism or acute renal failure (*p*: 00.3; value: 2.65 m/s; AUC ROC curve: 0.739; sensitivity: 73.3; specificity: 66.7; CI: 0.95, inferior: 0.613; superior: 0,866).

**Conclusions:**

Among hospitalized patients with COVID-19, echocardiography was normal in 51(46%) patients, and 20 (18%) patients presented with a decreased left ventricle ejection fraction. Tricuspid velocity was related to the composite endpoint of mortality, pulmonary thromboembolism or acute renal failure.

## Introduction

COVID-19 has become a pandemic during 2020 after an infectious outbreak observed in the city of Wuhan, China, in December 2019 [1–11]. In early May 2021, more than 158 million COVID-19 cases have been reported worldwide, with more than 3,3 million deaths and four countries (USA, India, Brazil, and Russia) have presented the most cases and related deaths (https://www.worldometers.info/coronavirus/#countries). In early May 2021, Brazil reported more than 15,1 million cases (third highest worldwide) and more than 421,000 deaths (second highest worldwide) (https://www.worldometers.info/coronavirus/#countries). COVID-19 diagnosis can be made using multiple approaches, such as genome sequencing using direct polymerase chain reaction (PCR) and virus isolation [1–11], and different imaging modalities (such as echocardiography and thoracic tomography) can add to the diagnosis of clinical complications. Complete understanding of the physiopathology of COVID-19 infection is yet to be achieved, but a substantial increase in cytokines is observed in patients affected by the virus [12–19]. Cardiovascular complications can be observed, such as myocarditis and myocardial dysfunction, pericarditis, myocardial infarction, thromboembolic events, and arrhythmias. Echocardiography can be used to add information concerning cardiac involvement, evaluate the extension of the complications related to COVID-19 infection and also provide prognostic information. To data, among different continents, few echocardiographic studies have been performed—some have performed complete echocardiographic studies, and others have used focused echocardiography [20–24].

Information is lacking concerning complete echocardiographic analysis of Brazilian COVID-19 in-hospital patients and the relationship between the echocardiographic findings and clinical events, such as mortality, thromboembolism and acute renal failure.

Therefore, we investigated the echocardiographic findings of COVID-19 in-hospital patients to predict the composite endpoint of mortality, pulmonary thromboembolism or acute renal failure.

## Methods

### Patients

The study comprised 111 of 154 consecutive adult (> 18 years old) symptomatic patients admitted from March to August 2020 to Hospital Israelita Albert Einstein, São Paulo, Brazil, with a positive diagnosis of COVID-19 (positive reverse transcription-polymerase chain reaction assay). Among the 154 patients, 43/154 (27.8%) patients were not included in the investigation because of poor quality of information. We performed complete transthoracic echocardiography and chest thoracic tomography, collected laboratory findings (biomarkers), considered the demographic data, comorbidities and clinical in-hospital events and related the findings to the composite endpoint of mortality, pulmonary thromboembolism or renal failure that occurred during hospitalization Echocardiographic examinations and thoracic computed tomography were performed following the request of the attending physician responsible for the patient, based on the patient’s clinical needs. Thoracic involvement of the parenchyma was considered as follows: > 25%, 25–50, and > 50%.

Acute renal failure was defined as any of the following: 1- Increase in serum Creatinine ≥0.3 mg/dL (≥26.5 μmol/L) within 48 h or; 2- Increase in serum Creatinine ≥1.5 times baseline, which is known or presumed to have occurred within the prior 7 days; or 3- Urine volume < 0.5 mL/kg/h for 6 h [25]. This investigation received the approval of the Research Ethics Committee of the Hospital Israelita Albert Einstein (CEP-HIAE) (Institutional Review Board, Comissão Nacional de Ética em Pesquisa No 3.960.096). All patients or a person legally responsible signed the informed consent forms for the echocardiographic examination.

### Echocardiography

The patients underwent complete transthoracic echocardiography under the request of the attending physician responsible for the patient, based on clinical needs. Echocardiography was performed following current international recommendations for COVID-19 echocardiographic performance [26]. Thus, to perform the echocardiographic examinations, personal protection equipment, including airborne protection (N-95 masks), gloves, head covers, face shield protection and fluid-resistant gowns were required. The echocardiographic examinations were performed in a standard manner employing the same equipment (Epiq 7; Philips Medical Systems, Bothell, WA, USA) by cardiologists with complete echocardiographic training. The acquisition of echocardiographic data was from bedside studies performed in COVID-19 intensive care units or internal ward units, with posterior offline analysis in the Echolab section at a workstation employing the software Q lab 13.0 (Philips Medical Systems, Bothell, WA, USA). The echocardiographic examination comprised a two-dimensional echocardiogram with colour Doppler, spectral Doppler and tissue Doppler, as well as longitudinal strain measurements derived from speckle tracking concerning the left and right ventricles. Imaging acquisition comprised longitudinal parasternal projections of the left, transverse and apical 2, 3 and 4 chambers, following the American Society of Echocardiography standardization [27]. To evaluate the left ventricular volumes and ejection fraction, we employed the Simpson biplane method using an automatic calculation algorithm. Diastolic function was analysed considering the evaluation of the spectral Doppler of the transmitral flow, measurements of the maximum velocities of the E and A waves, E/A ratio and E wave deceleration time, and tissue Doppler traces obtained from the apical four-chamber view, with the Doppler sample in the basal region of the ventricular septum (medial mitral annulus) and lateral mitral annulus. The E/e’ ratio was obtained from the septal and lateral segments of the mitral ring. The pulmonary artery systolic pressure was estimated by the tricuspid reflux. Cardiac valves were analysed using bidimensional echocardiography and Doppler and Colour Doppler techniques. Global longitudinal left ventricular strain was analysed from the apical planes of four, three and two chambers. To analyse the right ventricle, we evaluated the tricuspid annular plane systolic excursion (TAPSE), fractional area change (FAC), tissue Doppler (S wave) and longitudinal strain parameters. Longitudinal right ventricular strain was obtained from the apical plane of four chambers for better visualization of the right ventricle.

**Fig. 1:**
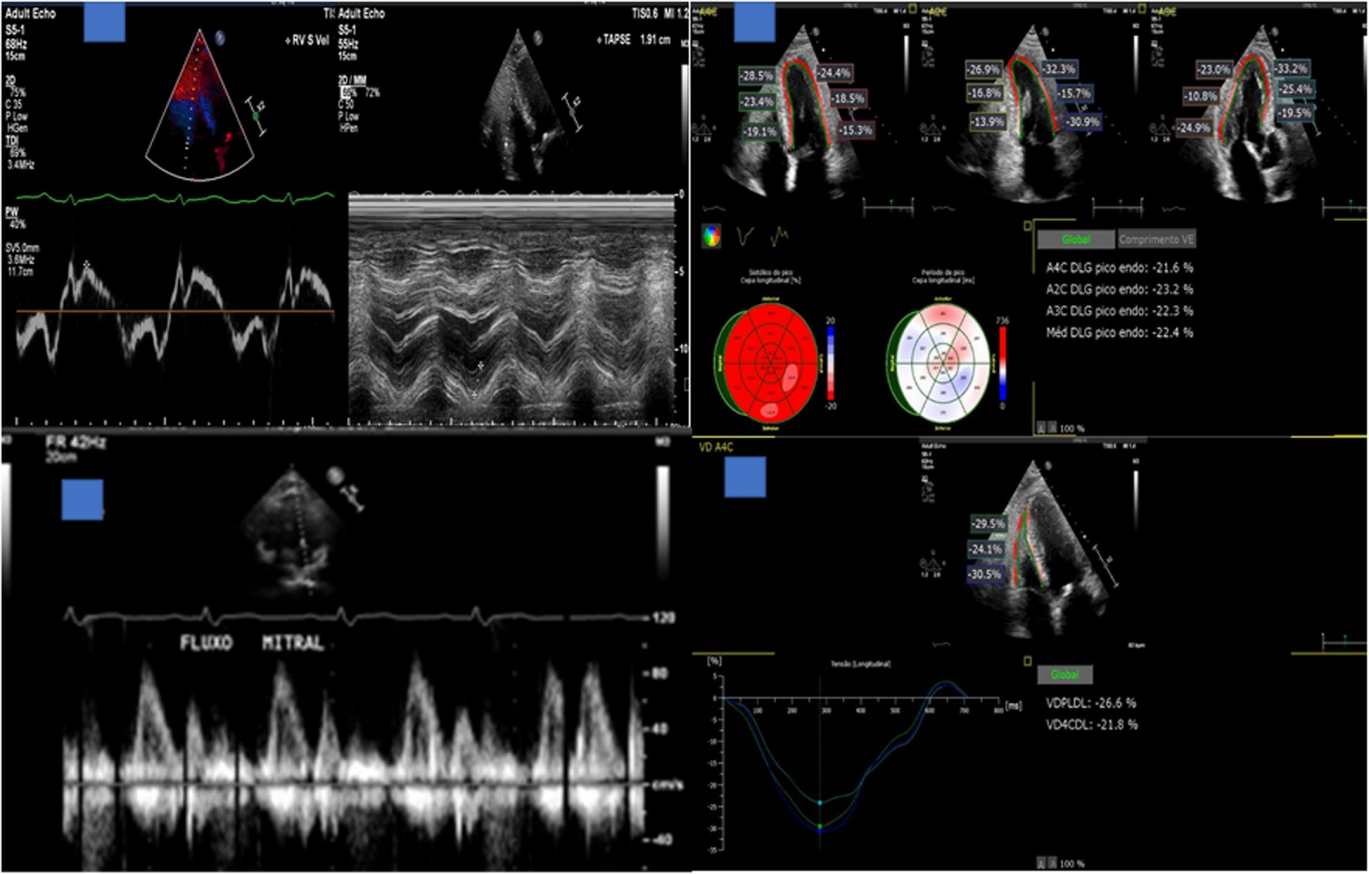
Images of the complete echocardiographic protocol of the investigation

**Fig. 2:**
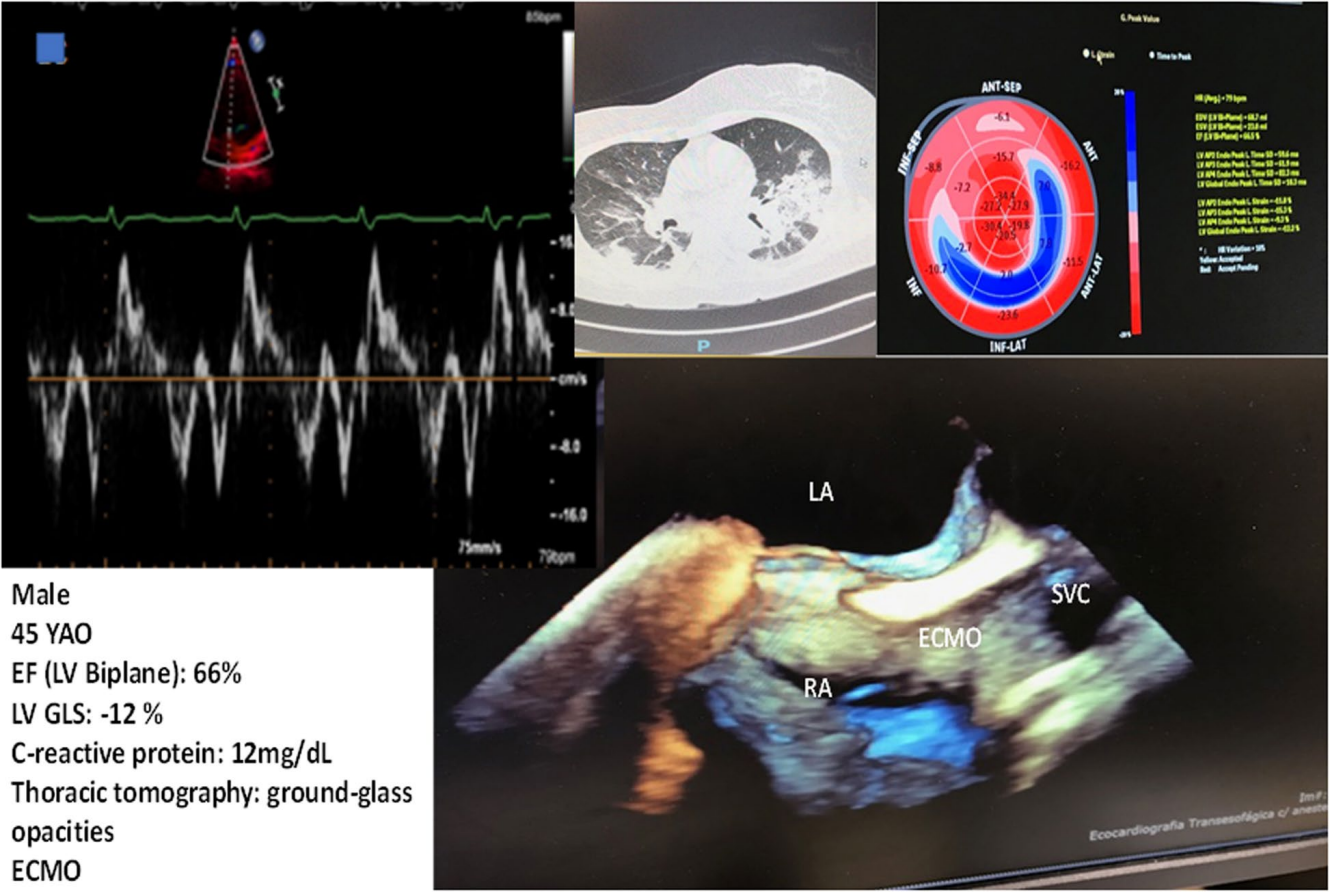
Images of a 45 year-old COVI 19 male patient presenting normal left ventricular ejection fraction (biplane EF: 66%), decreased longitudinal 2D global strain (− 12%), elevated C-reactive protein (12 mg/dL), ground-glass opacities on computed thoracic tomography, under ECMO therapy

Images from echocardiographic studies are depicted in Figs. 1 and 2.

**Fig. 3:**
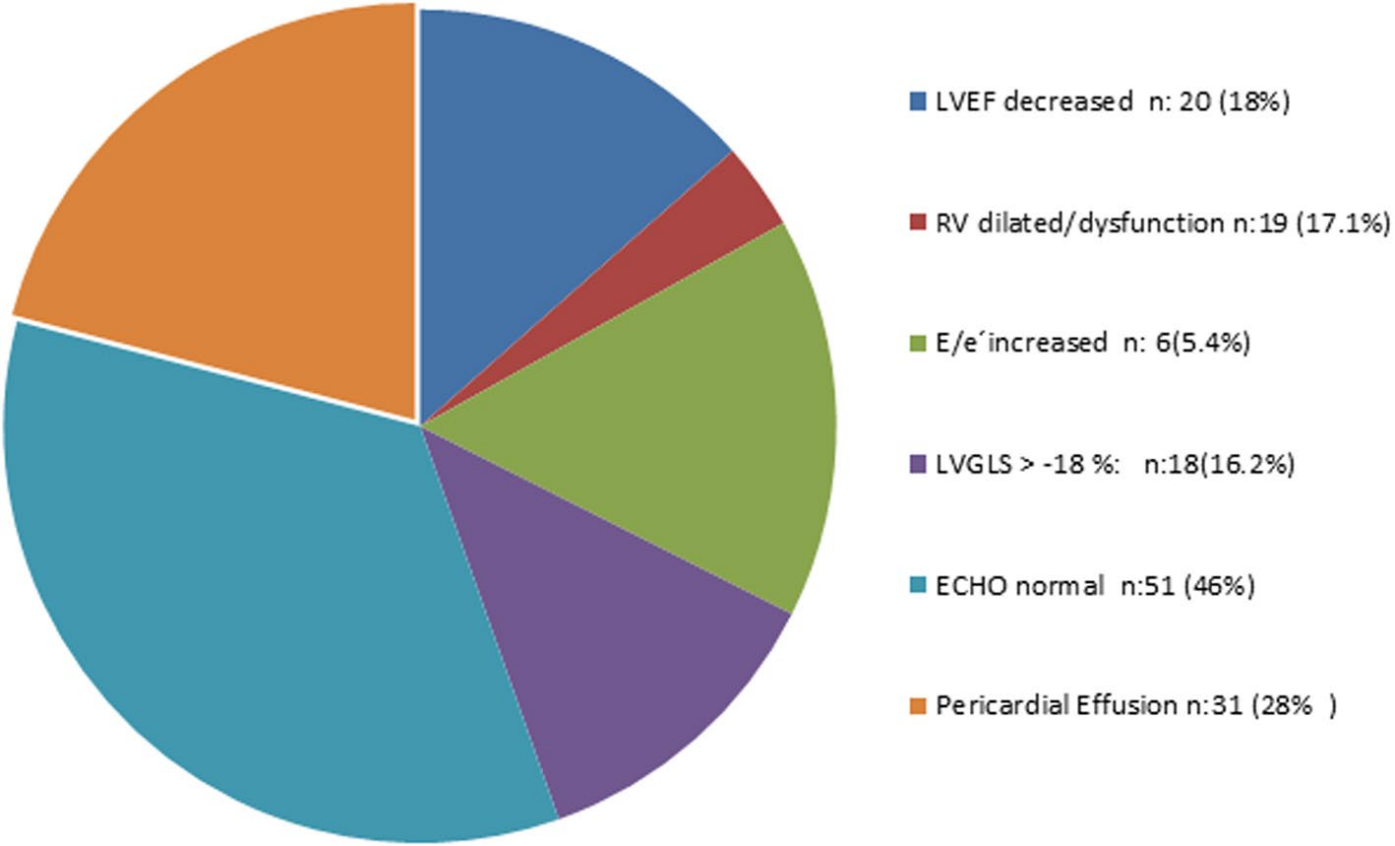
Distribution of the echocardiographic findings

Figure 3 depicts the distribution of the echocardiographic findings.

### Statistical analysis

The patients were analysed using qualitative parameters described as absolute and relative frequencies and quantitative parameters (mean, standard deviation, minimum and maximum) [28, 29] The associations between mortality and qualitative characteristics were evaluated using chi-squared test or exact tests (Fisher’s exact test or the likelihood ratio test); quantitative characteristics were compared between mortality or hospital discharge using using Student’s t-test or the Mann-Whitney test according the probability distribution of the variables [27, 28].

The area under the receiver operating (ROC curve) was constructed for echocardiographic parameters related to the prediction of a composite endpoint of mortality, pulmonary thromboembolism or acute renal failure of COVID-19 in-hospital patients, and odds ratios were calculated by logistic regression for the echocardiographic parameters and composite group of events. Analyses were performed using IBM-SPSS for Windows version 20.0 software, 5% significance level.

## Results

The clinical information, occurrence of comorbidities, clinical in-hospital evolution, complications related to COVID-19 infection, laboratory data, echocardiographic findings were observed in Tables 1, 2, and 3. Two hundred fifty-four (254) echocardiographic examinations (up to 8) were performed in 111 patients. A high rate of mortality (18.9%) was observed, with most of the patients (55.4%) remaining hospitalized for 7 to 20 days.

**Table 1:** Characteristics of COVID-19 in-hospital patients

Demographic information	N (%)
**Total No.**	111
**Age, (mean, SD, range), y**	67, 17 (19–101)
**Sex**	
Male	65(58.5)
Female	46 (41.5)
**Comorbidities**	
Diabetes	26 (23.4)
**Cardiovascular disease**	
Hypertension	38 (34.2)
Coronary artery disease	3 (2.7)
Congestive Heart Failure	3 (2.7)
Chronic Respiratory Disease	
Chronic obstructive pulmonary disease	11 (9.9)
Smoking	5 (4.5)
Kidney disease	7 (6.3)
Liver disease	1 (0.9)
Hypothyroidism	13 (11.7)
Neoplasms	8 (7.2)
**BMI**	
Obesity (> 30)	32 (28.8)
**Clinical evolution**	
Discharge	87 (78.4)
Still in-hospital	3 (2.7)
Death	21 (18.9)
**In-hospital period (days)**	
< 7	27 (25)
7–30	60 (55.6)
> 30	21 (19.4)
**Intubation and mechanical ventilation need**	48 (43.2)
**Intubation period (days)**	
< 7	7 (14.5)
7–20	29 (60.6)
20–30	5 (10.4)
> 30	7 (14.5)
**Clinical events (No: 54)**	
Myocardial infarction	10 (9.3)
Pulmonary Thromboembolism	7 (6.3)
Deep Vein Thrombosis	5 (4.5)
Renal Failure	12 (10.8)
Hemodyalisis	9 (9.8)

*BMI* body mass index (expressed as weight in kilograms divided by height in meters square), *COVID-19* coronavirus disease 2019

**Table 2:** Laboratory characteristics of COVID-19 in-hospital patients

Parameter	Mean	SD	Minimum	Maximum
Amilase, U/L	90.61	72.01	19	392
Creatine Phosphokinase, IU/L	325.7	1476.60	1.12	13,141
Creatinine, mg/dL	2.91	12.33	0.26	115
Lactate dehydrogenase, U/L	362.32	268.59	4.7	2500
Glucose, mg/dL	132.42	51.20	41	379
C-reactive protein, (NV < 0.3) mg/dL	50.28	78.67	0.3	444.3
Aspartate aminotransferase, U/L	144.60	771.84	14	7000
Alanine transaminase, U/L	129.59	692.27	9	7000
Blood urea nitrogen, mg/dL	68.60	65.31	12	357
Hemoglobin, g/dL	11.44	2.49	6	16.4
White blood cells, μL	2901.31	7513.77	3	54,480
Monocytes, cells/mL	629.93	576.46	2.548	2698
Lymphocytes, μL	1643.21	4397.32	1	44,674
Platelets,103/μL	250.45	110.57	29	601
D-dimer, (NV < 500) ng/L	2403.99	2291.71	1,31	7650
Fibrinogen, (NV: 200–400) mg/dL	533.70	853.89	138	7650
Troponin-I, (NV < 10) ng/L	97.19	168.60	5	968
Interleukin-6, (NV: 5–15) pg/mL	359.1	864.12	1.4	5000
BNP, (NV < 100) pg/mL	233.92	302.76	5	1578
Ferritin, (NV men: 20–500, women: 20–200) ng/mL	848.46	685.08	43.6	3187
Pro-calcitonin, (NV < 0,5) ng/mL	6.59	41.87	0.02	311
Lactate, (NV < 10) mg/dL	16.80	8.73	11	32

*NV* normal value, *COVID-19* coronavirus disease 2019

**Table 3:** Echocardiographic data in COVID-19 in hospital patients

Transthoracic Echocardiography	Mean	SD	Minimum	Maximum
LVEF (%)	58.20	8.18	16.4	70.6
LV Longitudinal Global Strain (%)	−21.01	3.79	−27.3	−13
RV Longitudinal Global Strain (%)	−15.69	3.90	−21.3	−6.8
RV Free Wall Longitudinal Strain (%)	−25.38	5.48	−36.5	−14.5
Left atrium (mm)	37.88	6.03	24	54
Septum (mm)	13.33	9.40	7	61
Left Ventricle Posterior wall (mm)	10.55	3.93	7	46
Left ventricle diastolic diameter (mm)	43.13	11.10	8	60
Left ventricle systolic diameter (mm)	32,03	7.14	22	55
RV diameter (mm)	27.51	7.30	18	74
Aortic root diameter (mm)	33.45	4.32	23	42
PASP (mmHg)	39.40	9.56	23	55
TAPSE (mm)	19.18	6.29	1.9	38
Tricuspid inflow velocity (m/s)	2.56	0.51	1.7	4.2
FAC (%)	41.26	10.23	8.1	60
RV diastolic 4 chamber area (cm^2^)	16.21	5.34	6.8	37
RV systolic 4 chamber area (cm^2^)	9.71	7.88	2.1	73
E/A wave ratio	1.08	0.49	0.4	2,9
Mitral valve deceleration time (ms)	233.09	76.15	99	494
E/e’ lateral ratio	9,69	3,48	4	17
E/e’ septum ratio	9,61	4,23	5	29.5
Pericardium effusion n (%)	31 (28)	–	–	–

*LV* left ventricle, *RV* right ventricle, *LVEF* left ventricle ejection fraction, *PASP* pulmonary artery systolic pressure, *TAPSE* tricuspid annular plane systolic excursion, *FAC* fractional area change, *E/e*' *ratio* protodiaslocic mitral valve inflow ratio (pulsed Doppler/Tissue Doppler)

We also observed elevated levels of biomarkers (D-dimer, C-reactive protein, Troponin-I, Interleukin-6, and BNP), fibrinogen and pro-calcitonin.

Most of the patients (65%) presented normal echocardiographic findings. However, 20 (18%) presented with decreased left ventricle ejection, 18 (16.2%) with abnormal left ventricle global longitudinal strain, 35 (31%) with diastolic dysfunction, 6 (5.4%) with an E/e’ratio > 14, 19 (17.1%) with right ventricle dilated/dysfunction, and 10 (9.87%) with pericardial effusion. The distribution of the echocardiographic findings of the COVID-19 in-hospital patients is presented in Fig. 3. We did not observe associations between the echocardiographic findings and COVID-19 in-hospital mortality or the elevation of the biomarkers.

**Fig. 4:**
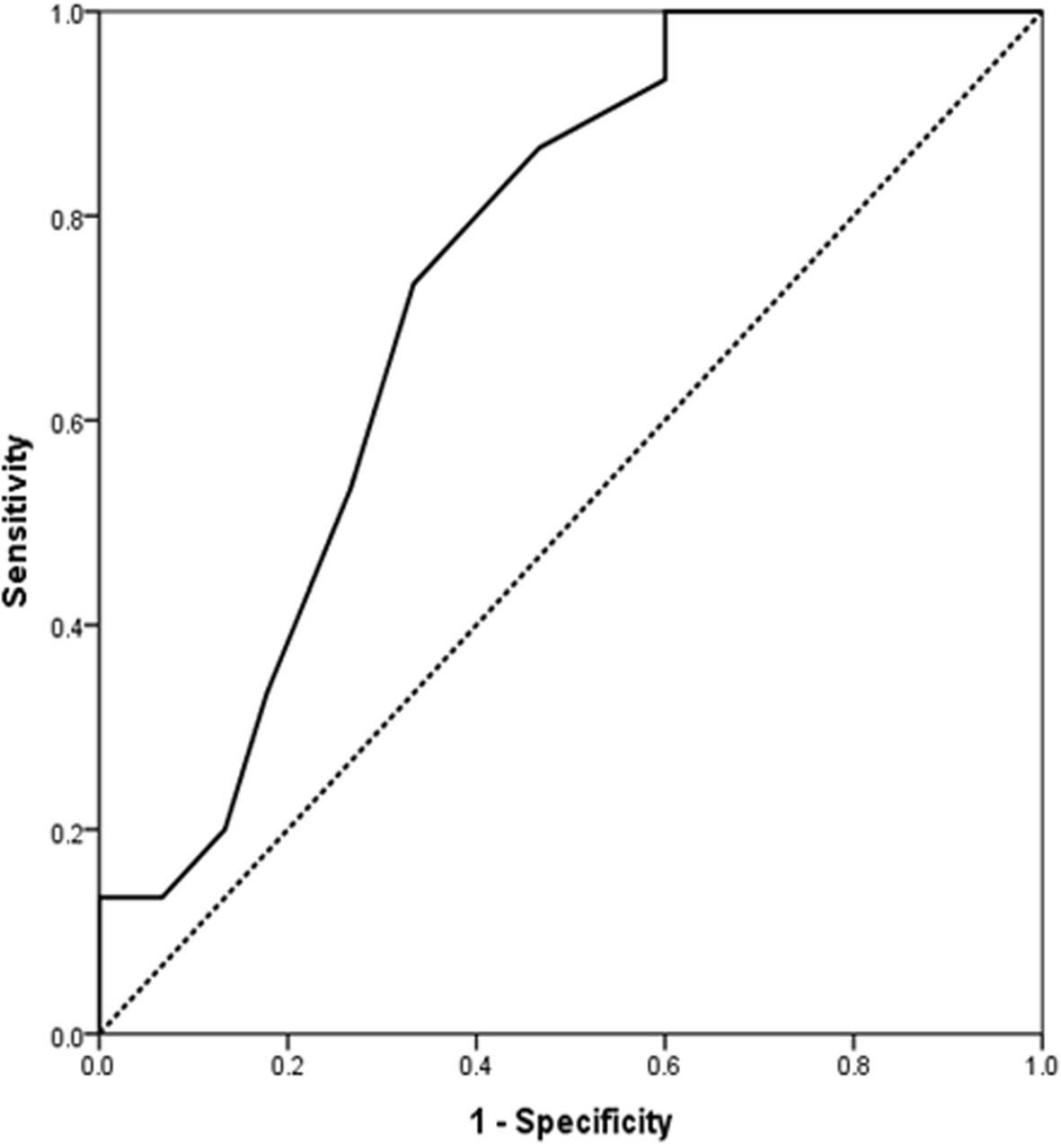
The area under the receiver operating characteristic curve of the tricuspid velocity for predicting a composite endpoint of mortality, pulmonary thromboembolism or acute renal failure for in-hospital COVID-19 patients

The echocardiographic findings related to the composite endpoint of mortality, pulmonary thromboembolism and renal failure are observed in Table 4. The tricuspid regurgitation velocity was the only echocardiographic parameter related to the composite endpoint of mortality, pulmonary thromboembolism and renal failure (*p*: 0.003). The area under the receiver operating characteristic curve of the tricuspid velocity for predicting a composite endpoint of mortality, pulmonary thromboembolism or acute renal failure for in-hospital COVID-19 patients is demonstrated in Fig. 4. For a tricuspid velocity of 2.65 m/s, the following values were obtained: AUC ROC curve: 0.739; sensitivity: 73.3; specificity: 66.7; CI: 0.95, inferior: 0.613; superior: 0,866; odds ratio: 5,55; IC (95%) (1.50–20.21, *p*:0.007). For a tricuspid velocity of 2.8 m/s (considered an important point for diastolic dysfunction), the values were as follows: AUC ROC curve: 0.633; CI: 0.95, inferior: 0.465; superior: 0,802; odds ratio: 3.14; IC (95%) (0.94–10.55, *p*:0.058). TAPSE presented marginal statistical significance for predicting the composite endpoint of mortality, pulmonary thromboembolism or acute renal failure (*p*: 0.064).

**Table 4:** Echocardiography related to the composite endpoint of mortality, pulmonary tromboembolism and renal failure

Echocardiography Parameter	
Parameter	P
Left Ventricle Ejection Fraction	0.709
LVGLS	0.865
RVFWLS	0.754
Left atrium	0.922
Septum	0.374
LV Posterior Wall	0.905
LVDD	0.845
LVSD	0.471
RV diameter	0.417
Aortic Root	0.582
PASP	0.392
TAPSE	0.064
FAC	0.851
RV Diastolic Area	0.695
RV Systolic Area	0.439
E/A Ratio	0.128
Mitral Valve DT	0.810
E/e' lateral ratio	0.505
E/e' septal ratio	0.129
Tricuspid regurgitation Velocity	**0.003**
**Student t test**	

*LV* left ventricle, *RV* right ventricle, *LVEF* left ventricle ejection fraction, *PASP* pulmonary artery systolic pressure, *TAPSE* tricuspid annular plane systolic excursion, *FAC* fractional area change, *DT* deceleration time, *DD* diastolic diameter, *SD* systolic diameter, *E/e*' *ratio* protodiaslocic mitral valve inflow ratio (pulsed Doppler/Tissue Doppler)

Chest computed thoracic tomography was performed in 54/111 (48.6%) patients. Thoracic involvement of the parenchyma was observed as follows: > 25% in 3/54 (5.5) patients, 25–50% in 46/54 (85.2%) patients, and > 50% in 5/54 (9.3%) patients. We did not observe an association between the tomography thoracic findings and mortality, elevation of the biomarkers or composite endpoint of mortality, pulmonary thromboembolism or acute renal failure or any complication after COVID-19 infection.

## Discussion

Thus far, few studies worldwide (none from Brazil) have addressed the role of echocardiography concerning COVID-19 infection [20–24, 30–32]. Some investigations have derived data from focused echocardiography [22, 23] and others have used complete echocardiographic examination [20, 21] information from a global online survey (69 countries worldwide) comprising an 11-item questionnaire completed on a smartphone [24]. One study enrolled patients prospectively [20], and others analysed retrospective information [22, 23]. The number of patients enrolled in the previous studies comprised 74 to 120 in the focused and complete studies [20–23], to 1272 patients in the global online survey relative to 17 days in April 2020 [24]. To our best knowledge, our study is the first to address the role of echocardiographic findings in COVID-19 in-hospital patients in Brazil considering thoracic tomography, biomarkers, comorbidities, and in-hospital clinical events and correlated the echocardiography findings for predicting a composite endpoint of mortality, pulmonary thromboembolism or renal failure. The high number of COVID-19 infections in Brazil (third highest worldwide, as of May 2021) makes those findings relevant. In our study, we prospectively analysed 111 patients who had undergone complete transthoracic echocardiographic examination.

The mean age of the patients in the previous focused and complete studies varied from 61 to 66 years, and 48 to 78% were men [20–23]. The age of the participants in the online survey was 62 (52–71) years, and 70% of them were men [24]. In our study, the mean age was 67 + years, but our population presented a wider age range (19–101 years), and 58.5% were men.

The mortality rate in our study was high (18.9%) compared with that in previous studies (2.3%), such as the summary report from the Chinese Centers for Disease Control and Prevention [12] a study from the Wuhan area (4.3% at a single centre in Wuhan) [10], or an investigation of 671 in-hospital patients from the Renmin Hospital of Wuhan University (9.3%) [13]. The mortality rate observed in our study was similar to that described in a study comprising 5700 patients from the New York City area (21%) [14], a study comprising 105 patients who had undergone focused echocardiography (20%) [22] and a study of 120 patients who undergone complete echocardiography (15%) [21]. However, our mortality rate was less than that reported in a retrospective study comprising 74 patients who had undergone focused echocardiography in Birmingham, United Kingdom (38%) [23]. The severity of COVID-19 infection in the United Kingdom study may be due to the rate of required intubation and mechanical ventilation (78%) [23] compared with that in other studies in which the role of echocardiography was reported (10–30%) [20–22]. In our investigation, the rate of required intubation and mechanical ventilation was high (43.2%), with an intubation period between 7 and 20 days for most patients (60%). Additionally, in our investigation, the systolic pulmonary artery pressure from the patients was slightly higher, ranging from 23 to 55 (mean: 39; median: 42) mmHg compared with other studies (median: 31 mmHg [21]; mean: 34 mmHg [23]).

An unfavourable outcome is expected in COVID-19 patients presenting hypertension, diabetes, previous cardiopathies, chronic pulmonary diseases, and immunosuppressive diseases [3, 6, 7, 10, 11]. In our investigation, the most common comorbidities were hypertension (34.2%) and diabetes (23.4%) compared with the rates of hypertension (37%) and diabetes (19%) in the online global survey [24]. The most common clinical event in our population during in-hospital hospitalization was renal failure (10.8%), followed by myocardial infarction (9%) and pulmonary thromboembolism in 7 (6.3%). In other investigations in which echocardiography was described, acute renal failure occurred in 13% and acute myocardial injury in 30.8% of the patients [21], and pulmonary thromboembolism occurred in 7% of the patients [23].

We observed marked elevation of biomarkers (C-reactive protein, D-dimer, BNP, troponin-I, and interleukin 6) and pro-calcitonin levels and a moderate increase in BNP. Other studies involving echocardiographic analysis, have reported an even higher increase in C-reactive protein (307 ± 114 mg/dl) but lower levels of D-dimer (657 (365–2066) ng/L) and troponin I (14 (6–67) ng/L) [22]. The elevation of biomarkers represents a very high inflammatory response due to virus infection and a very high procoagulative and prothrombotic state.

In our study, we observed normal echocardiographic examinations in 46% of the studies compared with 32% from an investigation employing complete echocardiographic investigation [20] and 44% from the global echocardiography survey [24]. The mean left ventricular ejection fraction was 58 ± 5% (mean), and the mean longitudinal global strain was − 21 ± 3.79%, although 18% of the patients presented with decreased LVEF compared with that in other studies (mean: 63 ± 7% [21], 57.9 ± 4% [20]; median: 55) [22]. In other study presenting 110 cases, it was reported decreased LVEF in 37% of the patients, 17% with RV dysfunction and 17% presenting biventricular dysfunction [29]. A focused echocardiography study comprising 749 patients described LV dysfunction in 37.8% of the patients and RV dysfunction in 13.7% [30]. Also in other investigation comprising 110 patients, 21.8% of the patients presented myocardial injury, and 54% from those patients LV dysfunction was demonstrated [32].. In other retrospective study comprising 68 patients, mortality was related to MAPSE [33]. For the analysis of the right ventricle, we found normal mean values for the parameters of TAPSE, FAC, and free wall longitudinal strain (although 2.7% of the patients presented abnormal values), but not for the analysis of the global longitudinal strain (mean: − 15.69 ± 3.90%), and 17.1% of the patients presented with right ventricular dilatation or abnormal function (considering one abnormal parameter). In other series, the right ventricular free wall longitudinal strain > − 23% was related to a prediction of mortality [21], dilatation of the right ventricle was associated with mortality [22], TAPSE< 17 mm and FAC < 35% were associated with higher levels of D-dimer and C reactive protein [34], right ventricular dilatation was associated with higher levels of troponin [24]. In 31 (28%) of the patients it was observed pericardial effusion, most of them (90%) just small. There was no evidence of tamponade. We observed no associations of the echocardiographic parameters and thoracic tomographic findings with COVID-19 in-hospital mortality or the elevation of the biomarkers.

However, the tricuspid velocity was related to the composite endpoint of mortality, pulmonary thromboembolism or acute renal failure (*p*: 00.3; value: 2.65 m/s; AUC ROC curve: 0.739; odds ratio: 5,55; IC (95%) (1.50–20.21, *p*:0.007). This observation is novel compared with other investigations concerning COVID-19 infection in which echocardiography was described [20–24]. This finding should be investigated in future series considering higher number of patients and should alert physicians concerning the elevation of pulmonary systolic pressure in COVID-19 patients.

### Limitations

This investigations concerning echocardiographic evaluation of in-hospital COVID-19 patients from a single Brazilian tertiary hospital centre, comprising a limited population, could have caused imbalances and bias in the analysis. Therefore, further studies enrolling multiple different hospitals will add important information concerning the role of echocardiography in this infectious scenario.

## Conclusion

Among hospitalized patients with COVID-19, echocardiography was normal in 51 (46%) patients and 20 (18%) patients presented with a decreased left ventricle ejection fraction. The tricuspid velocity was related to the composite endpoint of mortality, pulmonary thromboembolism or acute renal failure.

## Data Availability

Data and materials are at fully disposal.
